# A deep learning-based approach to automated rib fracture detection and CWIS classification

**DOI:** 10.1007/s11548-025-03390-5

**Published:** 2025-05-16

**Authors:** Victoria Marting, Noor Borren, Max R. van Diepen, Esther M. M. van Lieshout, Mathieu M. E. Wijffels, Theo van Walsum

**Affiliations:** 1 Trauma Research Unit, Department of Surgery, Erasmus MC, University Medical Center Rotterdam, Rotterdam, The Netherlands; 2 Department of Radiology and Nuclear Medicine, Erasmus MC, University Medical Center Rotterdam, Rotterdam, The Netherlands

**Keywords:** Ribfractures, Detection, Classification, CWIS

## Abstract

****Purpose**:**

Trauma-induced rib fractures are a common injury. The number and characteristics of these fractures influence whether a patient is treated nonoperatively or surgically. Rib fractures are typically diagnosed using CT scans, yet 19.2–26.8% of fractures are still missed during assessment. Another challenge in managing rib fractures is the interobserver variability in their classification. Purpose of this study was to develop and assess an automated method that detects rib fractures in CT scans, and classifies them according to the Chest Wall Injury Society (CWIS) classification.

****Methods**:**

198 CT scans were collected, of which 170 were used for training and internal validation, and 28 for external validation. Fractures and their classifications were manually annotated in each of the scans. A detection and classification network was trained for each of the three components of the CWIS classifications. In addition, a rib number labeling network was trained for obtaining the rib number of a fracture. Experiments were performed to assess the method performance.

****Results**:**

On the internal test set, the method achieved a detection sensitivity of 80%, at a precision of 87%, and an F1-score of 83%, with a mean number of FPPS (false positives per scan) of 1.11. Classification sensitivity varied, with the lowest being 25% for complex fractures and the highest being 97% for posterior fractures. The correct rib number was assigned to 94% of the detected fractures. The custom-trained nnU-Net correctly labeled 95.5% of all ribs and 98.4% of fractured ribs in 30 patients. The detection and classification performance on the external validation dataset was slightly better, with a fracture detection sensitivity of 84%, precision of 85%, F1-score of 84%, FPPS of 0.96 and 95% of the fractures were assigned the correct rib number.

****Conclusion**:**

The method developed is able to accurately detect and classify rib fractures in CT scans, there is room for improvement in the (rare and) underrepresented classes in the training set.

## Introduction

**Fig. 1:**
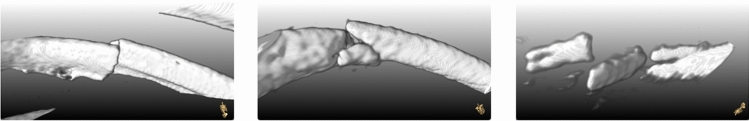
Volume renderings of typical fractures in CTs in the training set (classification according to the manual annotation). Left: simple, offset; middle: wedge, undisplaced; right: complex, displaced

Traumatic rib fractures are a common injury following thoracic trauma and are often caused by high force to the chest wall [15]. Rib fractures account for 10% of all trauma admissions with a prevalence of 10–40% among trauma patients [15]. These injuries result from high-energy trauma in younger patients, such as falls from heights or car accidents, and are frequently accompanied by other injuries. In elderly patients, they more often result from low-energy trauma [17]. In general, rib fractures lead to high morbidity and can cause mortality when combined with other conditions such as hemothorax, pneumothorax and soft tissue injuries [8]. The thoracic pain caused by rib fractures limits patients to cough and breathe deeply, which can result in atelectasis and pneumonia [14]. An increased number of fractures and older age are associated with increased rates of morbidity and mortality.

Rib fractures can be managed through surgical intervention or nonoperative treatment. A combination of optimal pain management, pulmonary physical therapy, oxygen suppletion and mechanical ventilation is considered essential for the nonoperative management of patients with rib fractures. Despite this treatment strategy, mortality and complications, such as pneumonia and acute respiratory distress syndrome, still occur often. Traditionally, patients were treated nonoperatively, nowadays more data are supporting the positive effects of early surgical stabilization of rib fractures (SSRF) [5]. SSRF aims to improve respiratory mechanics, reduce pain and prevent pulmonary complications by inserting rib fracture stabilizing systems. However, operative treatment increases the risk of surgical site infections with or without implant infections, potentially necessitating multiple additional surgical procedures.

For patients with multiple simple rib fractures, it remains unclear whether a nonoperative or surgical approach is more beneficial in terms of patient outcomes and cost-effectiveness, as well as which factors should influence this decision [16, 21]. It frequently occurs that these patients present with nonunion(s) of their rib fracture(s) a few months after the trauma. It is still a matter of debate if this patient group, with multiple simple (non-flail) fractures, could benefit from SSRF [6].

In 2020, the Chest Wall Injury Society (CWIS, www.cwisociety.org) published SSRF guidelines, including a rib fracture classification system. According to the CWIS classification [7], each fracture is classified with respect to three categories: fracture type, displacement and position. Fracture type can be simple, wedge or complex. Fracture displacement is either undisplaced (> 90% cortical contact), offset (< 90% cortical contact) or displaced (no cortical contact). Fracture location depends on the position of the fracture along the rib segment, either anterior, lateral or posterior. See also Fig. 1.

This classification system was conducted through a Delphi consensus study [7]. However, significant interobserver variability remains among clinicians in rib fracture classification using the CWIS classification, particularly concerning the type and displacement classification [18]. Implementation of SSRF guidelines is hampered by this inconsistency in rib fracture classification: it complicates communication in both clinical practice and scientific research. Given that rib fracture classification plays a critical role in decision-making, improving the reliability and accuracy of classification would be highly beneficial for optimizing treatment strategies. This enhancement would ensure that patients receive the most appropriate treatment through a standardized evaluation of their rib fractures.

Computed tomography (CT) is the most effective imaging modality for diagnosing rib fractures resulting from trauma and is the golden standard. Nevertheless, the literature shows that 19.2–26.8% of rib fractures are still missed during the diagnostic process, which is done manually by radiologists and other healthcare professionals [18]. Additionally, the manual classification of these fractures is time-consuming. To address these issues, a robust, (semi)automatic and reliable CT-based classification scoring approach is necessary.

The primary objective of this study therefore is to develop and evaluate a method for automated detection and classification of rib fractures according to the CWIS classification, including an approach for automated rib numbering. The contributions to achieve this objective are (1) the development of an automated rib fracture detection and classification approach, (2) the assessment of the method, including a comparison with intra- and interobserver variability and (3) an external validation of the method.

## Methods

**Fig. 2:**
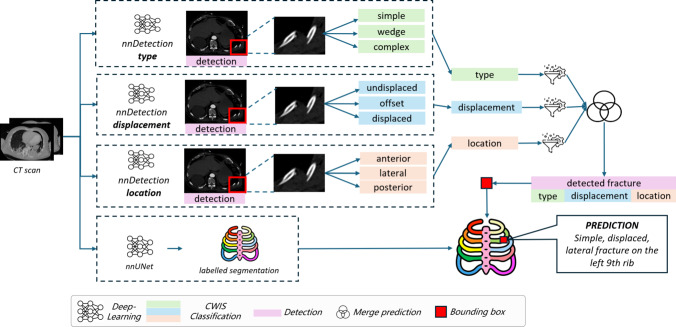
Overview of complete rib fracture detection and classification approach: three nnDetection networks are used to detect and classify fractures according to type, displacement and position; a separate nnU-Net is used to determine the rib number for each fracture

There are three components in the automated approach: (1) detection of the fracture, (2) classification of the fractures detected according to the CWIS classification and (3) assigning the rib number to the fraction. The overall scheme of the approach is shown in Fig. 2. For the detection and classification, we employ the nnDetection framework. The rib number classification is performed using a dedicated nnU-Net. Both approaches are described below.

### Detection and classification of rib fractures

We utilize nnDetection for the detection and classification of the fractures. It is a framework for semantic segmentations, which can also be utilized as an object detector, and follows the same self-configuring strategy as nnU-Net [3, 9]. Part of the framework consists of Retina U-Net. This merges the object detector RetinaNet, also used by one of the top five models in literature [23], with a U-Net architecture. Retina U-Net uses the feature pyramid network, which extracts features at different scales, enabling analysis of objects with varying sizes. To enhance the classification performance without introducing unnecessary complexity, Retina U-Net uses two additional layers dedicated to the classification task solely. This optimizes the model’s classification without making the model inefficient [10].

The nnDetection framework handles only one part of the classification at a time, for example, the fracture’s type. Therefore, the framework is used for training three separate models, each dedicated to one of the CWIS classifications. The reference standard for training the detection of fractures is scans with the fracture indicated by a spherical object with a radius of 10 mm, and their midpoint corresponding to the center of the rib fracture.

A trained nnDetection model produces bounding box coordinates, accompanied by probability scores, and one of the three labels for each detection. Consequently, one rib fracture detection with the complete CWIS classification can have three different bounding boxes. To address this, an ensemble approach is used by combining the results from the three models. First, the output of each individual nnDetection model is filtered to remove overlapping bounding boxes where the ones with the highest probability scores are kept. Two bounding boxes are considered overlapping if their Intersection-over-Union (IoU) score exceeds 50%. Secondly, the output of the models is merged with the requirement that there is an overlap of bounding boxes from at least two models, indicating the detection of a potential rib fracture by at least two models. These bounding boxes are combined through union. In cases where only two of the three models overlap, one of the labels cannot be assigned. In such instances, a label ’unknown’ is assigned.

### Rib numbering

For obtaining the number of the fractured rib, we need a segmentation with rib numbering. TotalSegmentator [20] is a recent segmentation tool that also generates segmentation labels per rib. Initial tests using this approach showed that it did not perform consistently, and gave errors in case of displaced ribs, or patients with eleven pairs of ribs (instead of twelve). We therefore decided to train a dedicated rib number labeling network. For this, we utilized nnU-Net. The training data were generated by using TotalSegmentator, and manually fixing the errors in the result using 3D Slicer.

**Table 1:**
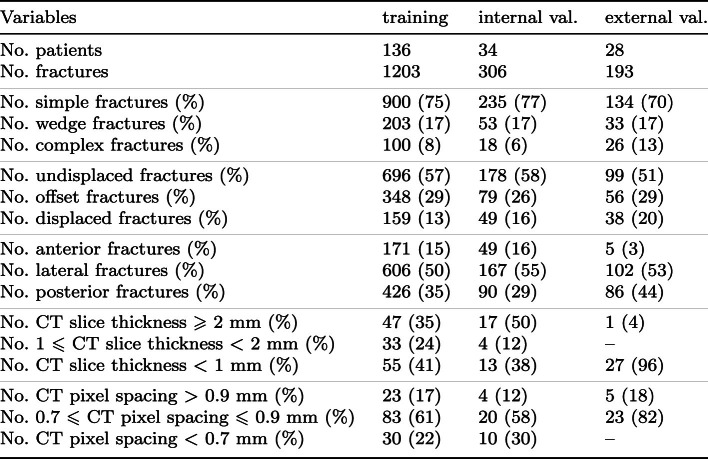
Data characteristics training and internal and external validation sets

### Combining classification and rib numbers

The last step combines the merged nnDetection models with the numbered rib segmentations. This is done by converting the bounding box coordinates into a binary label map. Subsequently, the binary label map and the numbered rib segmentations are compared. Bounding boxes that do not overlap with the numbered rib segmentations are discarded and for the others, the rib number is assigned to the fracture classification.

## Experiments and results

This section describes the experiments and results of our study. We first elaborate on the data used, briefly discuss implementation details and then detail the experiments performed.

**Table 2:** Interobserver agreement for the CWIS classification of rib fractures

Label	Cohens Kappa (95%CI)	Krippendorff’s Alpha (95% CI)	Interpretation
Type	0.74 (0.67–0.82)	0.76 (0.69–0.82)	Substantial
Displacement	0.82 (0.78–0.87)	0.82 (0.78–0.87)	Strong
Location	0.74 (0.69–0.80)	0.73 (0.68–0.79)	Substantial

**Fig. 3:**
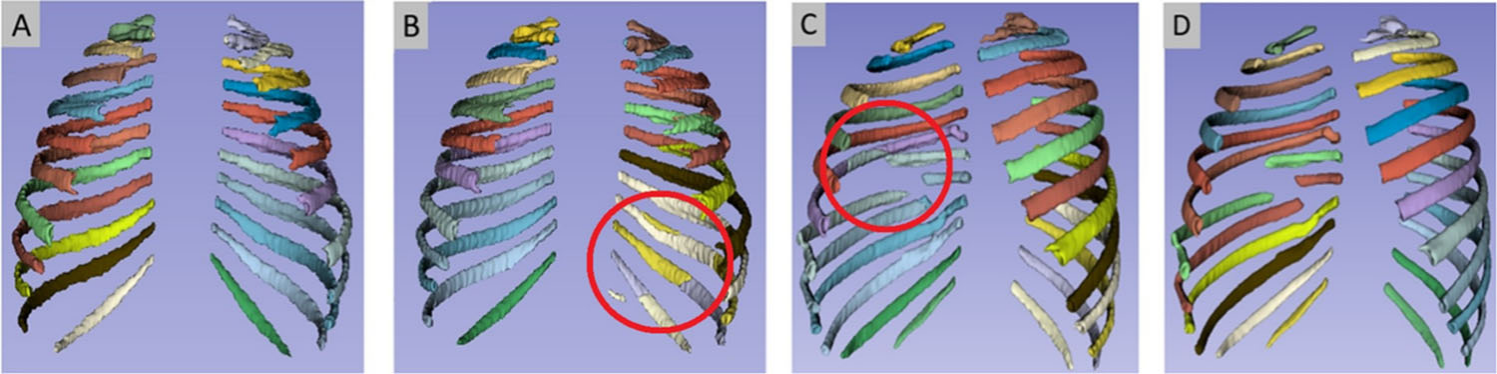
Examples of TotalSegmentator (**B**, **C**) and nnU-Net (**A**, **D**) rib number labelings where nnU-Net performs better

### Data

The data used consist of two different sets: one from Erasmus MC and from FixCon, a multicenter trial. The Erasmus MC data were retrospectively collected and anonymized CT scans of 100 randomly selected patients (1010 rib fractures) who were admitted to the Erasmus MC following blunt chest trauma. An ethical approval waiver was granted by the Medical Research Ethics Committee under the reference number MEC-2023-0039. All scans were acquired with a Siemens CT scanner. These scans had an in-plane image size of 512x512 pixels, with the number of slices ranging from 216 to 1513 slices.

All available FixCon patients who had a post-trauma thoracic CT scan that covered all ribs were included in our study. The FixCon dataset consists of data from 13 Dutch hospitals, and consisted of CT scans of 98 patients. Twenty-eight scans of this set, all from the same hospital not being the Erasmus MC, were held out of this set and used as external test set. Of the remainder of the 170 (100+70) scans, 34 were used for internal validation of the rib fracture detection and CWIS classification, including the rib numbering, and 136 were used for training the nnDetection models. These 136 training scans were also used to develop the rib numbering models: 106 scans were used for training the rib numbering, and 30 scans were used to compare the nnU-Net performance with the performance of TotalSegmentator. An overview of the data is provided in Table 1.

### Implementation

Training of the nnDetection and nnU-Net models was performed on the GPU cluster of the Erasmus MC with the 2090 Ti 11GB and Nvidia A40 48GB GPU’s. The inference time for a single CT scan is between 20 and 90 min per patient, depending on the number of slices. A random 80–20 train-test split was used on the number of fractures with the corresponding patients. To ensure class balance for the minority class, stratified sampling was performed. Furthermore, during training, a five-fold cross-validation strategy was implemented for each model. Both nnU-net (low-resolution 3D model) and nnDetection were used with their default settings,^1^ no hyperparameter tuning was applied.

### Reference standard and interobserver

The manual annotation (fracture detection and classification) process for obtaining the reference standard was conducted in software specifically developed for this project in MeVisLab. This semi-automatic tool stores each fracture’s coordinates, type, displacement, location and rib number.

The Erasmus MC dataset (100 CT scans) was annotated by a single researcher (NB). The FixCon dataset was annotated independently by two researchers (VM and MvD). In cases where there was disagreement on the type or displacement label, an experienced trauma surgeon (MW) solved the disagreement to obtain an accurate ground truth dataset. When one of the two labelers missed a fracture, the trauma surgeon made the final determination on whether a fracture was present. The classification of the fracture location (anterior/lateral/posterior) by VM was based on a measurement, while MvD’s classification was done subjectively. Therefore, VM’s classification is considered the truth for this category and disagreements in these cases were not solved by the experienced trauma surgeon.

In this dataset, there were 519 rib fractures noted by the two observers, of which 467 rib fractures were seen by both observers. Out of the 52 fractures detected by only one observer, 16 were identified by observer 1, while the remaining 36 were detected by observer 2. To evaluate interobserver agreement on fracture type, displacement and location, Cohen’s Kappa statistic was calculated based on the 467 fractures (Table 2). Additionally, Krippendorff’s Alpha was calculated to account for missing data. Disagreements were observed in 144 fractures for 162 classification tasks, with 40 disagreements in type classification, 52 in displacement classification and 70 in location classification.

**Fig. 4:**
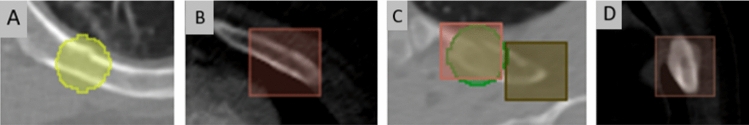
Example of a missed fractures (**A**), a true positive (**B**), a fracture classified as two fractures resulting in a false positive (**C**), a false positive due to callus formation (**D**). Squares indicate the detected fracture and circles indicate fractures labeled as the ground truth

**Table 3:** Characteristics of missed fractures (%) in internal (Int.) and external (Ext.) validation set, and sensitivity (sens., %) and precision (prec., %) of detection per fracture category for internal (Int.) and external (Ext.) data sets

	Simple	Wedge	Complex	Ant	Lat	Post	Undisp	Offset	Disp
Int. overall	82	15	3	24	58	18	66	8	26
Int. per class	22	17	11	30	22	12	23	6	33
Ext. overall	87	3	10	16	45	39	88	6	6
Ext. per class	20	3	12	100	14	14	27	5	4
Int. sens	95	36	25	79	94	97	90	80	61
Int. prec	87	47	40	93	93	93	89	69	95
Ext. sens	93	47	22	86	95	–	88	65	67
Ext. prec	83	43	83	95	86	–	81	63	86

**Fig. 5:**
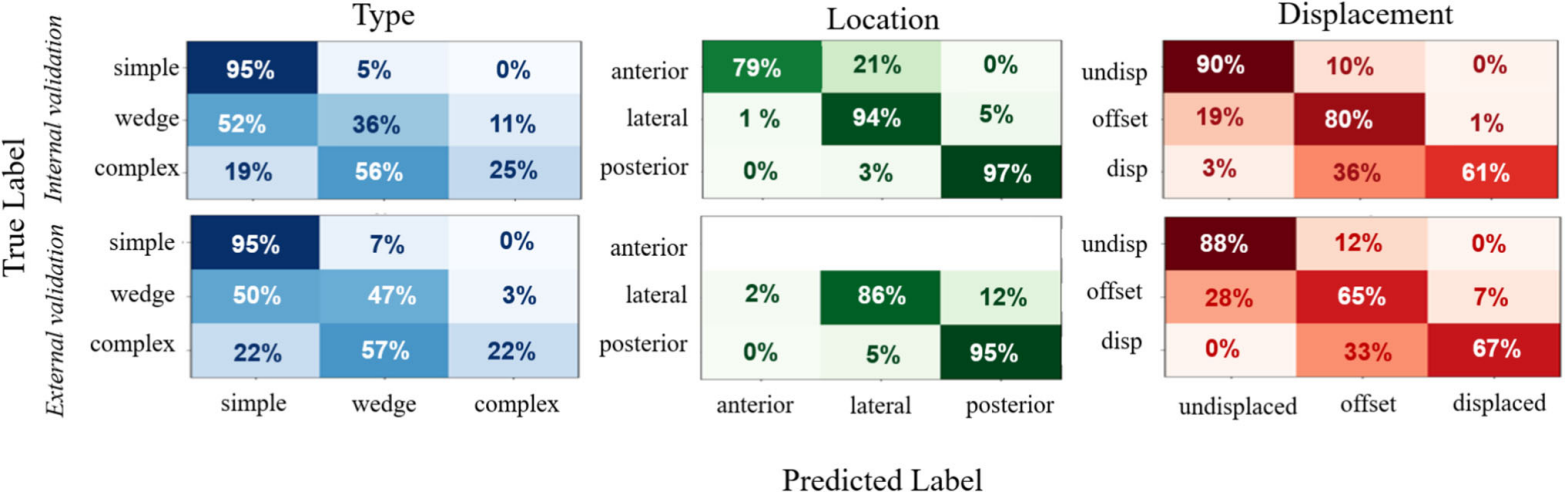
Confusion matrices for CWIS classification on internal (top row) and external (bottom row) validation set

### Rib number labeling

Our first experiment aimed to determine whether a dedicated nnU-Net can improve over TotalSegmentator, which already does rib number labeling. The assessment was performed manually in 3D Slicer, evaluating the following aspects by visual inspection: (1) is the correct rib number assigned; (2) does the segment represent a single rib; and (3) in the case of severely dislocated fractures: do the fragments have the same label? The assessment was done on the validation set of 30 CT scans (see Sect. 3.1), after training the nnU-Net with the semi-automatically obtained reference standard on 106 scans. A total of 716 ribs (30 scans) were evaluated, including two cases where only 11 pairs of ribs were present. Of these 716 ribs, 246 ribs were fractured. TotalSegmentator assigned the correct rib number to 92.5% of all ribs and 95.5% of the fractured ribs. In comparison, nnU-Net assigned the correct rib number to 95.5% of all ribs and 98.4% of the fractured ribs. There was at least one incorrectly labeled rib in seven patients when using nnU-Net, of which five were labeled incorrectly by TotalSegmentator too. TotalSegmentator had at least one error in eleven patients. Figure 3 illustrates two cases where nnU-Net had a better labeling compared to TotalSegmentator.

### Internal validation

To assess the performance of the model, the rib fracture detection, CWIS classification and rib number labeling (using nnU-Net) performance on the internal test set were assessed. The model achieved a detection sensitivity of 80%, a precision of 87% and an F1-score of 83% on this test set, with a mean false positive per scan (FPPS) of 1.11. The threshold for fracture detection was chosen such that the detection sensitivity on the training set was 82% (human scoring results). This sensitivity surpasses the range typically observed among clinicians, which falls between 73.2% and 80.8% [2, 4, 18, 22]. All classification labels were successfully assigned, as no cases involved only two overlapping nnDetection models. Examples of detection results are in Fig. 4. Missed fractures are frequently undisplaced fractures with a hardly visible interruption of the cortical bone (Fig. 4A). False positives were old fractures, indicated by callus formation around the fracture (Fig. 4D) or locations where the anterior rib transitions to the costal cartilage, due to irregularities in the cortical bone in this region, or fractures with displacement that were labeled as two fractures (Fig. 4C). A qualitative evaluation of the detection of rib fractures revealed three additional true positives that were not labeled in the ground truth (Fig. 4B). Table 3 presents the characteristics of the missed fractures and the fraction per class that was missed. Figure 5 illustrates the classification performance per class of all detected fractures; Table 3 contains the sensitivity and precision of the detection per classification category.

To assess the impact of CT parameters, we compared the detection and classification results for CTs with a slice spacing < 2 mm to those with a slice spacing $\ge$ 2 mm. For detection, precision, sensitivity and F1-score, these were, respectively, 83%, 81% and 82% and 90%, 79% and 84% for the < 2 mm and $\ge$ 2 mm slices, with both a FPPS of 1.1. There were not statistically significant differences in detection or classification results between both groups.

**Table 4:** Recent methods for rib fracture detection

	Year	Patients	Fractures	Sensitivity (%)	FPPS
Li et al. [11]	2023	18,172	–	93	0.5
Azuma et al. [1]	2023	539	4906	84	2.71
Zhou et al. [23]	2022	640	2853	95	0.17
Wang et al. [19]	2022	9265	43,803	85	0.35
Niiya et al. [13]	2022	918	–	93	1.9
Meng et al. [12]	2021	8829	34,699	92	0.14

Of the 244 detected fractures by nnDetection, 94% were assigned the correct rib number. One of the patients with mislabeled ribs had eleven pairs of ribs, leading to one fracture being assigned to the wrong rib number. In two other patients, there were artifacts (scattering) in the CT scan, resulting in four misclassifications.

### External validation

Lastly, the model was applied to the CT scans from the external validation set; Table 1 shows the data characteristics of this external validation dataset. The detection sensitivity on the external validation dataset was 84%, with a precision of 85%, an F1-score of 84% with a FPPS of 0.96 (27 in total); five false positives appeared to be fractures in a retrospective analysis. In clinical practice, we foresee that the results will still undergo clinical visual assessment, and false positives will be detected. Table 3 presents the characteristics of the missed fractures and the percentage of missed fractures per class. Figure 5 illustrates the classification performance per class of all detected fractures in a confusion matrix, Table 3 contains the sensitivity and precision of the detection per classification category. Of the detected fractures, eight were assigned an incorrect rib number, resulting in a rib number classification accuracy of 95%.

## Discussion

This study developed and assessed a model for detection, CWIS classification and rib number labeling of rib fractures. To accomplish this, three nnDetection models, responsible for automatic fracture detection and CWIS classification, were trained with a multicenter dataset. Additionally, a rib number labeling method was developed and evaluated.

The detection sensitivity of the model was 80% on its internal test set, with a precision of 87%, an F1-score of 83% and a mean FPPS of 1.11. This detection sensitivity is at the upper limit of the range observed for clinicians, which spans from 73.2% to 80.8% [2, 4, 18]. Missed fractures are most often simple undisplaced fractures. Besides the fact that these fracture types occur most often, simple undisplaced fractures can be subtle and may not show major changes in bone structure, and therefore do not create distinctive features that are easily detectable.

Classification performance remains challenging, particularly for accurately identifying the type of fractures. Label imbalance may cause the model to become biased toward the more prevalent classes (simple fractures), leading to suboptimal classification for less frequent fracture types (complex and wedge). A significant proportion of the underrepresented complex and wedge fractures are misclassified as simple, leading to low sensitivity for these fractures and reduced precision for simple fractures.

The automatic rib number labeling showed good performance, with 94% and 95% of rib fractures being assigned the correct rib number in the internal and external test sets, respectively. The results indicate that the presence of fractures does not necessarily lead to a higher rate of mislabeling, suggesting robust performance even in cases with fractures.

The external validation results confirm the strong potential of the model, with performance metrics slightly better than the internal test set. The label distribution of the external validation set is similar to the label distribution of the internal test set, see Table 1. However, the fraction of complex and posterior fractures is larger in the external validation dataset and lower for anterior fractures, compared to the internal test set. The increase in detection performance on the external validation set thus may be attributed to the fact that complex and posterior fractures are detected more often, which results in higher detection sensitivity for the external validation dataset, since it contains relatively more complex and posterior fractures, compared to the internal test set. Also, the results of the external validation (containing CT scans from a hospital that is not in the training set) show that the model has been trained on a dataset that is representative for the Dutch situation. Application to other countries or populations would require additional assessment.

Similar strategies, but with different classes for classification, such as displaced, non-displaced, buckle and old fractures, have been developed by others. Most of these use common object detection approaches, either in 3D, such as faster R-CNN [1], and a pretrained two-stage 3D object detector [13], or in 2D with subsequent merging [11, 13], or U-shaped based approaches, such as VRB-Net [12] or a U-net [19]. A comparison with these works (see Table 4) suggests that expanding the dataset may lead to increased sensitivity and precision in detection. Note that these approaches do not perform a CWIS classification. To improve the performance of the model, future efforts should focus on augmenting the dataset with underrepresented fracture classes and obtaining more annotations. This would help refine the model’s ability to accurately classify these types of fractures as well.

The processing time (20–90 min) does not limit the model’s applicability in acute care settings, where surgery within 24–72 h is desired for rib fractures. Also, in non-acute settings, the model could improve detection sensitivity and support clearer communication. Both may lead to earlier and more tailor-made treatment of patients with rib fractures. Moreover, a speed up could be obtained by preselecting the thorax-slices from the CT scans, and running the models in parallel.

Future research should investigate the impact of automated rib fracture detection and classification systems on the clinical decision-making processes of surgeons and other medical specialists, with a focus on changes in decision-making patterns. Additionally, it would be valuable to evaluate how the integration of these systems influences clinical outcomes.

## Conclusion

In this study, we developed and evaluated a method for automatic rib fracture detection and classification. Results on the internal and external validation set were similar, and at the level of the interobserver results. Specifically, the detection sensitivity and precision were 80% and 87%, respectively, with an 1.11 FPPS and an accuracy of 94% in rib number labeling. Future research should improve classification of underrepresented classes, and assess the value of these classifications for supporting therapeutic decision-making, e.g., via an outcome prediction model.

## Footnotes

1 For nnU-net version 2.5.1 was used, and the nnDetection version can be installed via -e git+https://github.com/MIC-DKFZ/nnDetection.git@98eb97f15d9f9d107fac11d27c153e18932ab635#egg=nndet.
